# Serum Amyloid P Component Binds Fungal Surface Amyloid and Decreases Human Macrophage Phagocytosis and Secretion of Inflammatory Cytokines

**DOI:** 10.1128/mBio.00218-19

**Published:** 2019-03-12

**Authors:** Nicole E. Behrens, Peter N. Lipke, Darrell Pilling, Richard H. Gomer, Stephen A. Klotz

**Affiliations:** a Department of Immunobiology, University of Arizona, Tucson, Arizona, USA; b Department of Biology, Brooklyn College, Brooklyn, New York, USA; c Graduate Center of the City University of New York, New York, New York, USA; d Department of Biology, Texas A&M University, College Station, Texas, USA; e Department of Medicine, University of Arizona, Tucson, Arizona, USA; University of Texas Health Science Center; Scripps Institute; Humanitas University

**Keywords:** SAP, cytokines, functional amyloid, innate immunity, macrophage polarization

## Abstract

In patients with invasive fungal diseases, there is often little cellular inflammatory response. We tested the idea that binding of the human constitutive plasma protein serum amyloid P component (SAP) (also called PTX2) to Candida albicans dampens the innate immune response to this fungus. Many pathogenic fungi have cell surface amyloid-like structures important for adhesion and biofilm formation. Human SAP bound to fungi that expressed functional cell surface amyloid, but SAP had minimal binding to fungi with reduced expression of cell surface amyloid. In the absence of SAP, phagocytosis of fungi by human macrophages was potentiated by expression of amyloid on the fungi. SAP binding to fungi inhibited their phagocytosis by macrophages. Macrophages pretreated with SAP displayed reduced fungal phagocytosis, reduced secretion of inflammatory cytokines (IFN-γ, IL-6, and TNF-α), and increased secretion of the anti-inflammatory cytokine IL-10. SAP bound to fungi or added to the medium upregulated the expression of the anti-inflammatory receptor CD206 on macrophages. These findings suggest that SAP bound to amyloid-like structures on fungal cells dampens the host cellular immune response in fungal diseases such as invasive candidiasis.

## Abstract (precis)

Macrophages are a key part of our innate immune system and are responsible for recognizing invading microbes, ingesting them, and sending appropriate signals to other immune cells. We have found that human macrophages can recognize invading yeast pathogens that have a specific molecular pattern of proteins on their surfaces: these proteins have structures similar to the structures of amyloid aggregates in neurodegenerative diseases like Alzheimer’s disease. However, this surface pattern also causes the fungi to bind a serum protein called serum amyloid P component (SAP). In turn, the SAP-coated yeasts are poorly recognized and seldom ingested by the macrophages, and the macrophages have a more tolerant and less inflammatory response in the presence of SAP. Therefore, we find that surface structures on the yeast can alter how the macrophages react to invading microbes.

## INTRODUCTION

In humans, the pentraxin serum amyloid P component (SAP) is constitutively present in serum (typically ∼30 µg/ml) (1, 2). SAP binds to amyloid fibrils derived from precursors, including serum amyloid A, immunoglobulin light chain, and apolipoprotein A1, and also binds to pathogenic viruses (3) and bacteria (4, 5). The binding of SAP to microorganisms may be an important factor in determining the outcome of some disseminated infectious diseases. For example, one report demonstrated that bacteria to which SAP bound were not opsonized or phagocytosed efficiently (5). In mouse models of disseminated infection with Escherichia coli, Streptococcus pyogenes, or Neisseria meningitidis (all of which bound SAP), the bacteria were lethal when injected intravenously. SAP knockout mice survived infections with similar numbers of the same bacteria, but when SAP knockout mice were injected with microbes and human SAP, the mice succumbed to disseminated infection (5). Thus, the presence of SAP can increase virulence in bacterial infection models.

SAP avidly binds to amyloid and is a prominent and invariant constituent of all extracellular amyloid deposits (6). We have proposed that SAP also binds to fungi because of functional amyloid on fungal cell surfaces (7). Candida albicans cell surface adhesins form amyloid-like nanodomains (8). These amyloid nanodomains form in response to shear stress and are essential for cell-cell aggregation and biofilm formation (9). The amyloid interactions are functional, in the sense that amyloid-forming ability is an important part of adhesin activity and is evolutionarily conserved in fungal adhesins and some bacterial adhesins (10). We previously observed SAP on fungi in invasive *Candida* in human tissue and found that SAP binding to the fungi, in part, required the presence of functional amyloid on the surfaces of fungal cells (11). We also observed SAP on fungal surfaces in deep-seated fungal diseases, including aspergillosis, coccidioidomycosis, and zygomycosis (12).

Polymorphonuclear leukocytes (PMNs) and macrophages are sparse in anatomic sites of invasive candidiasis even in cases with normal or elevated white blood cell counts (11, 13). Marked reductions of host immune cells were also seen in deep-seated disease with *Aspergillus,* zygomycetes, and *Coccidioides* (12). Other investigators have also noted the profound absence of host immune cells in the vicinity of invasive fungi including aspergillosis and mucormycosis, again in spite of normal or elevated peripheral white blood cell counts (14–16). Thus, leucopenia alone cannot explain the lack of cellular infiltrates. Pentraxin 3 and C reactive protein (CRP), two pentraxins generally acknowledged to be proinflammatory, were not detected on *Candida* species invading gastrointestinal tissue (13). This apparent lack of an innate immune response is reminiscent of the histology of extracellular amyloid deposits, with their absence of any detectable cellular response even as the amyloid destroys organs (17). A commonality between these conditions is SAP binding to amyloid in extracellular deposits or amyloid on fungal surfaces.

Here we report that SAP binding to C. albicans leads to decreased phagocytosis by human macrophages and to a less aggressive host innate cellular response. These changes may be a molecular explanation for the histological findings of minimal inflammation in invasive fungal diseases.

## RESULTS

### SAP binds to yeast cells in proportion to the amount of functional amyloid expressed on the fungal surface.

**FIG 1:**
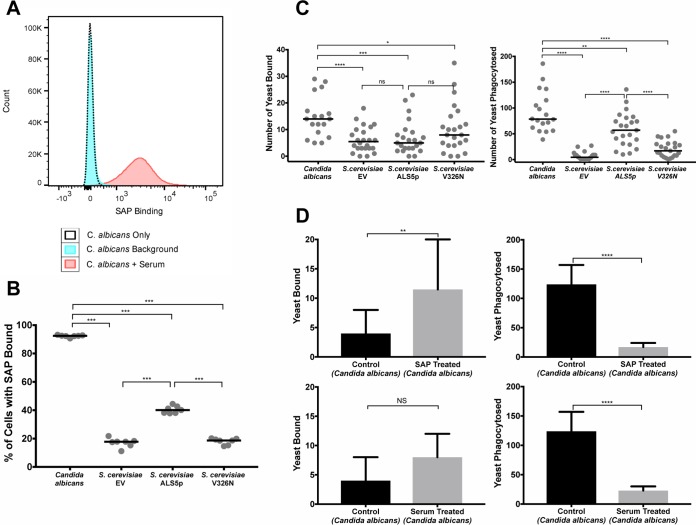
Binding and phagocytosis of yeasts by human macrophages. (A) SAP binding to C. albicans yeast cells determined by SAP antibody labeling and flow cytometry. (B) SAP binding to yeast cells of C. albicans, S. cerevisiae-EV, the amyloidogenic S. cerevisiae*-*Als5p^WT^, and the nonamyloidogenic, S. cerevisiae*-*Als5p^V326N^. SAP was offered to yeasts in normal human serum. Each symbol is the average of one of seven independent experiments. (C, left) Binding of fungi to macrophages upon the addition of C. albicans, S. cerevisiae*-*EV, S. cerevisiae*-*Als5p^WT^, or S. cerevisiae*-*Als5p^V326N^. (Right) Phagocytosis of the same strains of yeast cells by macrophages. Each symbol is the average from three independent experiments. (D) Effect of SAP on macrophage binding and phagocytosis of C. albicans. (Top left) Binding of yeast cells to control macrophages or macrophages pretreated with SAP. (Top right) Phagocytosis of C. albicans by control macrophages or macrophages pretreated with SAP (30 µg/ml). (Bottom left) Binding of yeast cells in serum-free medium and yeast cells offered SAP in normal human serum to macrophages. (Bottom right) Phagocytosis by macrophages of yeast cells in serum-free medium and yeast cells offered SAP in normal human serum (results of three separate experiments each with 3,200 macrophages counted/variable). Note that the *y* axis is 10-fold higher for phagocytosis than the binding graphs. Statistical significance was determined by Mann-Whitney U test and indicated as follows: ns, not significant; *, *P* < 0.05; **, *P* < 0.01; ***, *P* < 0.001; ****, *P* < 0.0001. Medians are plotted to show central tendency. Bars represent 95% confidence intervals.

To elucidate SAP binding to C. albicans, yeast cells were exposed to SAP from normal human male AB serum by incubating yeasts in serum for 1 h (the most physiological method of providing SAP, avoiding the problem of SAP aggregation [18]). Figure 1A shows a flow cytometric profile demonstrating that most C. albicans cells bound SAP and there was minimal background fluorescence. We then compared SAP binding to C. albicans to SAP binding to nonpathogenic laboratory strains of Saccharomyces cerevisiae. S. cerevisiae W303-1B transformed with an empty vector (pJL1) (hereinafter, S. cerevisiae-EV) expresses low levels of endogenous amyloid, as does a transformant that expresses the nonamyloidogenic Als5p^V326N^ form of the C. albicans adhesin Als5p (S. cerevisiae-Als5p^V326N^) (19). Heterologous expression of the amyloidogenic adhesin Als5p^WT^ in S. cerevisiae (S. cerevisiae-Als5p^WT^) causes yeast cells to adhere and form biofilms in a manner similar to that of C. albicans (20, 21). Figure 1B shows that a median of 92% of C. albicans bound SAP. By comparison, only 18% of S. cerevisiae-EV cells bound SAP; S. cerevisiae*-*Als5p^V326N^, 19% of cells; the amyloidogenic S. cerevisiae*-*Als5p^WT^, 40% of cells. Thus, SAP bound to yeast cells in the order of their expression of cell surface amyloid: C. albicans > S. cerevisiae*-*Als5p^WT^ ≫ S. cerevisiae*-*Als5p^V326N^ or S. cerevisiae-EV.

### SAP binding to C. albicans is inhibited by the calcium chelator EDTA.

SAP binding to amyloid fibers is calcium dependent, and bound SAP is dissociated from amyloid fibers with EDTA (18). To determine whether SAP binding to C. albicans is also sensitive to EDTA, we added EDTA to mixtures of serum and C. albicans. EDTA significantly inhibited SAP binding to the cells (Table 1) (see Fig. S1A in the supplemental material). These data suggest that SAP binding to C. albicans is at least partly dependent on calcium.

**TABLE 1:** Inhibition or augmentation of yeast binding of SAP and phagocytosis by human macrophages in the presence or absence of various additives^*a*^

Treatment	% of yeasts binding SAP (mean ± SEM)	No. of yeasts phagocytosed (mean ± SEM)	% inhibition or augmentation^*b*^	Significance^*c*^
Control^*d*^	74 ± 2			
10 mM EDTA	67 ± 2		−9	0.08
25 mM EDTA	61 ± 1		−18	0.005
50 mM EDTA	53 ± 1		−28	0.001
100 mM EDTA	33 ± 1		−55	<0.001

Phagocytosis, control^*e*^		136 ± 9		
100 mM mannose		30 ± 9	−78	<0.001

Phagocytosis, control^*e*^		90 ± 4		
CRP		104 ± 4	+16	0.024

Phagocytosis, control^*e*^		112 ± 9		
SAP-soaked yeast		43 ± 3	−62	0.004

Phagocytosis, control^*f*^		53 ± 7		
SAP (30 µg/ml)		14 ± 2	−74	<0.001
SAP *ex* serum^*g*^		9 ± 1	−83	<0.001

a All values are from a minimum of three independent experiments, each with a minimum of eight replicates.

b Inhibition shown by negative values, and augmentation indicated by positive values.

c Significance is for comparison of variable with macrophages only. Significance was determined by Mann-Whitney U test or unpaired t test.

d Results of flow cytometry; serum, yeasts, and additive incubated for 1 h. See Fig. S1 in the supplemental material for a graphical representation of the experiment.

e Treatments added in serum-free medium to 96-well plates followed by the addition of yeasts, shaking at 37°C, and then counting phagocytosed cells.

f Results from growth of macrophages in chamber slides.

g SAP *ex* serum indicates SAP offered in serum.

10.1128/mBio.00218-19.1 FIG S1 Phagocytosis of C. albicans yeasts by human macrophages under different treatment conditions. Download FIG S1, DOCX file, 0.2 MB. Copyright © 2019 Behrens et al. 2019 Behrens et al. This content is distributed under the terms of the Creative Commons Attribution 4.0 International license.

### Phagocytosis of fungi is dependent on expression of fungal functional amyloid.

We investigated the phagocytosis of yeasts by human macrophages and found that macrophages bound approximately twice as many C. albicans cells as S. cerevisiae cells. The three S. cerevisiae strains bound in similar numbers (Fig. 1C, left). However, the macrophages ingested significantly different numbers of the yeasts (Fig. 1C, right). Macrophages phagocytosed a median of 79 C. albicans yeast cells/50 macrophages and 57 cells/50 macrophages for the amyloid-bearing strain S. cerevisiae*-*Als5p^WT.^ In contrast, few non-amyloid-bearing yeasts were ingested: S. cerevisiae-EV, 4 cells/50 macrophages and S. cerevisiae*-*Als5p^V326N^, 17 cells/50 macrophages. These data suggest that the presence of yeast cell surface amyloid increases the phagocytosis of yeast cells.

### SAP treatment of macrophages inhibits macrophage phagocytosis of yeast cells.

Having shown that SAP binding to yeast cells and macrophage phagocytosis of yeast cells are both increased by the presence of yeast surface amyloids, we investigated the effect of SAP on macrophage phagocytosis of yeast cells. Human macrophages were incubated with or without SAP for 60 min in 96-well plates, the wells were washed with serum-free medium, and then yeasts were added for 30 min in serum-free medium. The SAP pretreatment led to an ∼3-fold increase, from ∼4 to ∼11 C. albicans cells bound per 50 macrophages (Fig. 1D, top left), but a 86% reduction in macrophage phagocytosis, from a median of 124 yeast cells phagocytosed/50 macrophages in control wells (no SAP) to 17 yeast cells/50 macrophages in wells with SAP (Fig. 1D, top right). In contrast, pretreating macrophages with the related pentraxin, C-reactive protein (CRP) (50 µg/ml) enhanced yeast phagocytosis by 16% (Fig. S1B). CRP bound to negligible numbers of yeast cells on flow cytometry (7%). SAP from Millipore contained low concentrations of azide and EDTA; both of the additives at various concentrations were incubated with macrophages to determine whether these additives affected phagocytosis, and they did not (Fig. S1C). Furthermore, phagocytosis results were not affected using unaltered SAP from Millipore or SAP that had been desalted (removing any trace of azide, EDTA, and NaCl) (Fig. S1B and C). Together these data support the hypothesis that SAP treatment of the macrophages inhibits phagocytosis of C. albicans.

**FIG 2:**
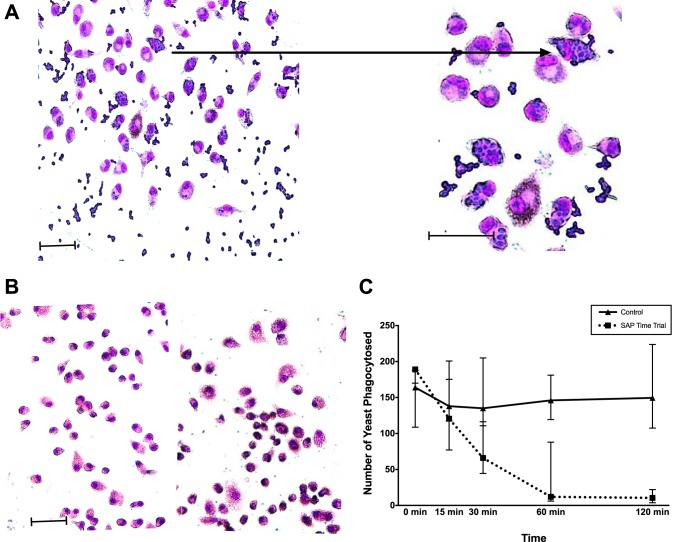
Phagocytosis of yeast. (A) Macrophages in serum-free medium (pink cytoplasm, ∼40 μm in diameter) and untreated C. albicans yeast cells (blue ovals, ∼2 to 3 μm existing in singlets and doublets and multiples of the same). Many yeasts are phagocytosed by macrophages. Bar, 50 µm. (Upper right) Close-up of macrophage that has phagocytosed 15 yeast cells. Bar, 50 µm. (B) Photomicrograph of macrophages pretreated with SAP. Note the round conformation of the macrophages compared to those in panel A above. Bar, 50 µm. (C) Phagocytosis of yeasts by macrophages preincubated in serum-free medium in the presence or absence of SAP (30 µg/ml). Results represent yeasts phagocytosed by 400 macrophages per time point. Results at zero and 15 min are not significantly different. The time points of 30, 60, and 120 min are significantly different with *P* values of <0.05, <0.01, and <0.01, respectively. Medians are depicted to show central tendency. Bars represent 95% confidence limits, and significance was determined by the Mann-Whitney U test.

Only 10% to 40% of macrophages phagocytosed yeast (Fig. 2A). The morphology of SAP-treated macrophages was noticeably different from that of control macrophages: the former were smaller and more compact with rounded cell contours rather than pseudopodal extensions. This may be an attribute of the immunological quiescence of the cells due to SAP treatment (22, 23). A time course showed that SAP treatment of the macrophages was rapid, with >90% inhibition of phagocytosis after 60 min of incubation with exogenously added SAP prior to the addition of yeasts (Fig. 2C). Because mannose is an inhibitor of SAP binding to macrophages (24), a major saccharide found in the cell wall of fungi, and the mannose receptor found on macrophages is important in yeast phagocytosis, we tested the effect of D-mannose on the interaction of yeasts and macrophages. Phagocytosis of yeasts was inhibited by 78% in the presence of 100 mM mannose (Table 1), implying involvement of the macrophage mannose receptor.

### Serum or SAP treatment of yeast inhibits macrophage phagocytosis of yeast.

To determine whether incubation of yeast with serum affects macrophage phagocytosis of yeast, Candida albicans yeasts were incubated in normal human serum for 1 h, washed, and then incubated with human macrophages. Compared with pretreatment of the macrophages with SAP, serum treatment of the yeasts did not significantly increase binding of yeasts to the macrophages (Fig. 1D, bottom left). Nevertheless, serum treatment of the yeasts decreased macrophage phagocytosis of yeast by 82% (Fig. 1D, bottom right). This result is not significantly different from the 86% inhibition of phagocytosis following pretreatment of macrophages with SAP. The same phenomenon occurred with macrophages cultured on chamber slides (Table 1). In addition, macrophage phagocytosis of yeast decreased by 62% when the yeast cells were presoaked in purified SAP for 1 h in TBS-C (Table 1 and Fig. S1D). These results indicate that pretreatment of yeast or macrophages with SAP profoundly inhibits macrophage phagocytosis of yeast. The results were consistent for SAP added as a constituent of serum or as purified SAP in buffer.

### The combination of SAP and *Candida* increases expression of two macrophage M2 markers.

**FIG 3:**
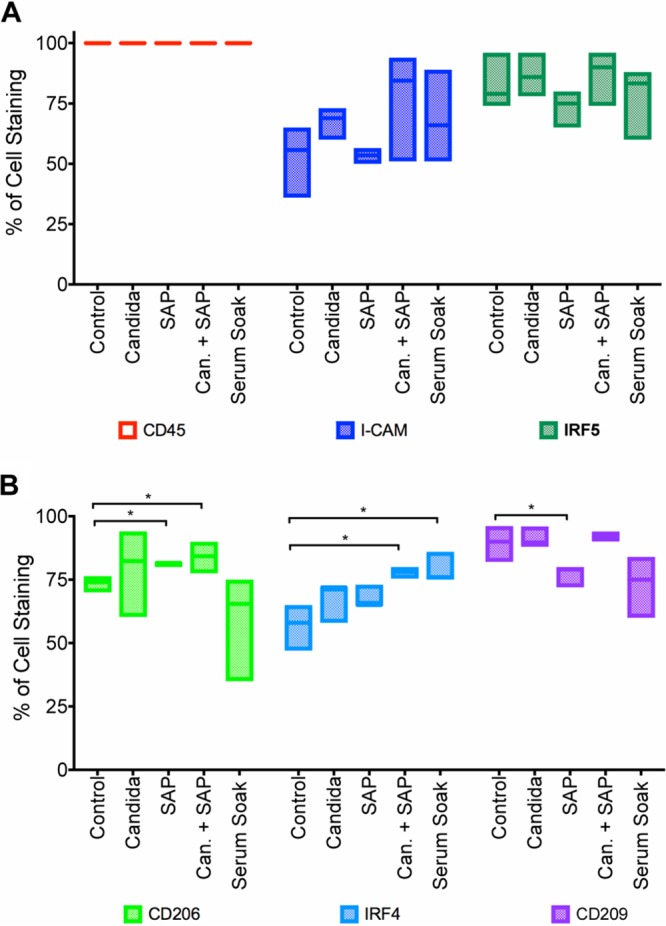
Expression of markers by macrophages. (A and B) Macrophages were incubated in serum-free medium (control) or in serum-free medium plus the following: C. albicans alone; SAP alone; SAP and C. albicans; or C. albicans yeast cells offered SAP in normal human serum. Results show the percentage of macrophages staining for markers following different treatments. A bar within a colored box is the median value, and the box represents the 95% confidence intervals of values from four separate donors. Values that are significantly different (*P* < 0.05) by Mann-Whitney U test are indicated by a bar and asterisk.

Macrophages change markers and physiology when exposed to different external signals (25). We assessed the effects of SAP and yeasts on macrophage markers associated with polarization: ICAM-1 (CD54), CD206, CD209, and the transcription factors IRF4 and IRF5. All macrophages showed expression of CD45, demonstrating their origin from the bone marrow (Fig. 3A). There were no significant effects of *Candida*, SAP, or serum on the percentage of cells expressing the two M1 markers ICAM-1 and IRF5 (Fig. 3A). Examining the correlation between yeast phagocytosis and marker expression, we observed that all macrophages that phagocytosed yeasts expressed ICAM-1. SAP and *Candida* plus SAP increased the percentage of cells expressing the M2 marker CD206, and *Candida* plus SAP and serum-soaked *Candida* increased the percentage of cells expressing IRF4 (Fig. 3B). SAP decreased the percentage of cells expressing the M2 marker CD209 (Fig. 3B). Together, these results indicate that SAP and *Candida* can affect some marker expression in macrophages.

### Compared to *Candida* alone, SAP and *Candida* decrease accumulation of the proinflammatory cytokine IFN-γ and increase accumulation of the anti-inflammatory cytokine IL-10.

**FIG 4:**
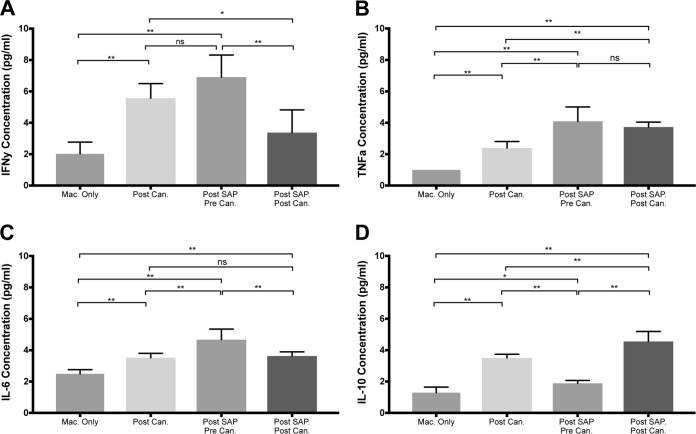
Cytokine secretion by macrophages after various treatments. The treatments were : macrophages in serum-free medium (Mac. Only), addition of C. albicans to macrophages (Post Can.), addition of SAP only (post SAP Pre Can.), and pretreatment of macrophages with SAP (30 µg/ml) followed by the addition of C. albicans (Post SAP. Post Can.). Medians are plotted to show central tendencies with 95% confidence intervals. Statistical significance was determined by Mann-Whitney U test and indicated as follows: ns, not significant; *, *P* < 0.05; **, *P* < 0.01.

Macrophage signaling through secreted cytokines is a key event in innate and acquired immunity. Therefore, we assessed extracellular cytokine accumulation in the presence of C. albicans yeasts and/or SAP. Macrophage extracellular accumulation of IL-6, IFN-γ, TNF-α, and IL-10 was increased by either yeasts or SAP (Fig. 4). However, compared to SAP alone or yeast alone, combining SAP and yeast decreased the accumulation of the inflammatory cytokine IFN-γ and increased the anti-inflammatory cytokine IL-10 (Fig. 4). Extracellular IL-17A accumulation was increased when yeasts were added to the cell culture, and SAP did not significantly affect the accumulation of this cytokine (Table 2).

**TABLE 2:** IL-17A secretion by macrophages in response to different treatments^*a*^

Treatment	IL-17A level (pg/ml)^*b*^	Significance^*c*^
Macrophages only	1.1 ± 0.1	
Yeast cells added to macrophages	2.5 ± 0.4	0.006
SAP (30 µg/ml) added to macrophages	1.0 ± 0.1	NS
SAP (30 µg/ml) + yeast cells added to macrophages	3.7 ± 0.3	0.002

a IL-17A secretion by macrophages in response to addition of SAP and/or yeasts for two replicates in each experiment (three different experiments).

b Values are means ± standard errors of the means from three experiments.

c Significance was determined by Mann-Whitney U test. NS, not significant.

### SAP does not inhibit PMN phagocytosis of yeasts.

**FIG 5:**
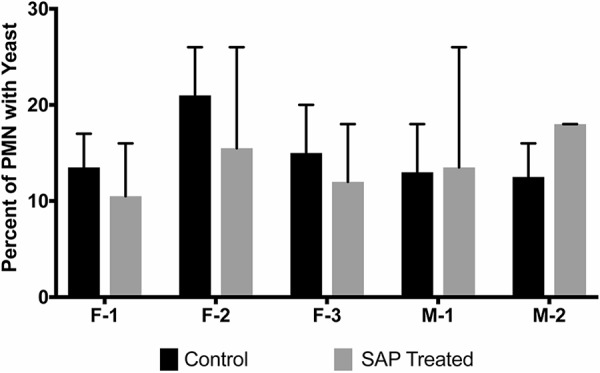
Phagocytosis of C. albicans yeasts by human polymorphonuclear leukocytes (PMNs). Controls are PMNs offered yeasts in serum-free medium; SAP treated are PMNs pretreated with SAP (30 µg/ml), washed, and offered yeasts in serum-free medium. Cells were from three females (F-1 to F-3) and two males (M-1 and M-2). Medians are plotted to show central tendency with 95% confidence levels. There was no significant difference with the Mann-Whitney U test between controls and SAP-pretreated PMNs with respect to phagocytosis.

Because PMNs are important in cellular innate immunity, we examined what effect SAP has on PMN phagocytosis of C. albicans. Human PMNs were pretreated with SAP (30 µg/ml) for 60 min, and C. albicans yeasts were then added in serum-free medium. After 30 min of coincubation, there was no significant effect of SAP on PMN phagocytosis of yeast, and this effect was seen for both male and female donors (Fig. 5).

## DISCUSSION

Results reported here may explain the highly attenuated inflammatory response in infected tissue, a paradoxical feature of invasive fungal disease in humans (11, 13–16). In several invasive fungal diseases, fungal cells in deep infections display surface amyloid-like structures and are coated by the amyloid-binding SAP (11–13). We now report that a consequence of SAP binding to fungi is the down-modulation of the innate cellular immune response.

### Human macrophages recognize fungal surface amyloid-like structures.

Macrophages bind yeasts through lectin receptors, including the mannose receptor (MR) (CD206) and DC-SIGN or CD209 (26, 27) to facilitate phagocytosis even when bacteria or yeasts are unopsinized (27, 28). Our data reflect the involvement of CD206, because phagocytosis was significantly inhibited by the addition of D-mannose. Under the conditions of our experiments, phagocytosis of yeasts was clearly related to the level of surface amyloids present on the fungi, but how much occurs through CD206 or DC-SIGN is unknown. Macrophages were largely indifferent to our laboratory strain of S. cerevisiae which expresses very low background levels of the amyloidogenic proteins Flo1 and Flo11 (29), and there was minimal phagocytosis of yeast expressing the nonamyloidogenic Als5p^V326N^ (19). In contrast, yeast cells expressing the wild-type amyloidogenic sequence were phagocytosed at high levels. C. albicans yeast cells were phagocytosed to an even higher degree, and these cells possess a higher level of surface amyloid and a large number of potentially amyloidogenic adhesins (9, 10, 30). Therefore, formation of amyloid-like nanodomains could activate phagocytosis by clustering CD206 into high-avidity patches on the cell surface, as we have shown for activation of fungal adhesins (9). Bacterial functional amyloids have frequently been implicated as virulence factors in many diseases including involvement with innate immune cells (31). One example is the amyloid pilus of Mycobacterium tuberculosis that facilitates invasion of macrophages (32), and SAP markedly reduces M. tuberculosis phagocytosis (33). The phagocytosis of M. tuberculosis is similar to that of C. albicans, i.e., both are engulfed within a phagolysosome, the difference is that the bacteria persist in macrophages, whereas C. albicans is killed (34), or the fungus kills the macrophage after shifting to hyphal morphology (35). Phagocytosis of M. tuberculosis or Mycobacterium smegmatis in the presence of SAP also skews the macrophages toward the “tolerant” M2c cell type, and this skew can be reduced in the presence of small molecules that block SAP binding (36). Thus, in both microbes (M. tuberculosis and C. albicans), SAP may bind to amyloid-like structures and lead to increased microbe survival and propagation.

### SAP binding to fungi is dependent upon fungal expression of functional amyloid and is antiphagocytic.

The importance of SAP binding to bacteria and the implications this phenomenon might have on host defenses were reported years ago (5), but they have not been pursued systematically. It was proposed that binding of SAP to beads is opsonic, but SAP binding to bacteria protects the bacteria from degradation and is antiopsonic and anti-immunogenic (5, 37). Binding of SAP to C. albicans may give rise to similar antiphagocytic and anti-inflammatory effects. Wild-type C. albicans expresses a number of cell surface functional amyloids, the best studied is Als5p (19), a member of a family of amyloidogenic adhesive proteins variably expressed on the cell surface (7). In invasive candidiasis, SAP binds to functional amyloid nanodomains on the surface of the fungi. The consequences of this binding include inhibition of macrophage phagocytosis and reduction in secretion of inflammatory cytokines. Thus, human SAP, a soluble pentraxin that binds avidly to pathological human amyloids (38), also binds to C. albicans functional amyloids.

The presence of functional amyloids explains the binding of SAP to S. cerevisiae-Als5p compared to S. cerevisiae*-*EV and S. cerevisiae-Als5p^V326N^, which has little surface amyloid. The SAP binding capacities of the yeasts were C. albicans *>* S. cerevisiae*-*Als5p^WT^ ≫ S. cerevisiae*-*Als5p^V326N^ > S. cerevisiae*-*EV, an order consistent with the ability of yeasts to form amyloid-like surface nanodomains (9, 10, 39). The low levels of SAP binding could be due to residual surface amyloids (39) or exposure of phosphoethanolamine and/or phosphocholine in the yeast cell wall, as they are ligands of SAP (40–42). It is possible that SAP binding to microbial amyloid is common: SAP binding to surface proteins of *Neisseria meningitidis* was investigated, and two of the three identified SAP-binding proteins, NMB0667 and NMB201 (43), have multiple regions with high amyloid potential, like many fungal adhesins (data not shown).

SAP coating of fungi markedly inhibited phagocytosis, with a corresponding increase in C. albicans bound but not engulfed. The results were similar whether the yeasts or macrophages were treated with purified SAP or SAP was offered to yeasts in normal serum, and the effects of SAP on macrophage phagocytosis were detected within minutes. This SAP binding may result from fungal exploitation of SAP’s role as an immunomodulatory molecule, rather than as a human adaptation to infection. Fungal surface amyloids have evolved as components of biofilms, and SAP binding may be a fortuitous consequence (8, 9, 11, 23, 38).

### SAP is anti-inflammatory.

The consequences of SAP binding included reduction in accumulation of proinflammatory cytokines and an increase in accumulation of anti-inflammatory IL-10. IL-10 secretion by macrophages increased in response to SAP treatment of the macrophages or the yeast cells. These results confirm well-established anti-inflammatory properties of SAP, which has been previously shown to reduce neutrophil adhesion (44, 45), to inhibit maturation of monocytes to fibrocytes in tissue (44), and to coat cell debris which is scavenged by macrophages (37). Accordingly, SAP also directs macrophage differentiation toward resolving macrophage class M2c (22). The anti-inflammatory effect is dependent upon the production of IL-10 which follows SAP binding to DC-SIGN (46).

Our incubation of SAP with macrophages in fluid phase or bound to C. albicans yeasts led to changes in macrophage surface antigen expression. Markers of the M1 phenotype (ICAM-1, IFR5) were unchanged, whereas IRF4 and the M2 marker phenotype CD206 increased. Detection of CD209 (DC-SIGN) decreased with exposure to SAP. CD209 is a macrophage receptor that may account for up 30% of C. albicans phagocytosis (28), and SAP binding to DC-SIGN leads to increased IL-10 secretion that potentiates an anti-inflammatory response (46). Detection of DC-SIGN with antibodies was lower on the macrophages incubated with SAP, either due to downregulation of the receptor, or perhaps the antibody binding site was masked by the binding of SAP to the receptor. These results are in conformity with the anti-inflammatory properties of SAP (23) and along with the changes in cytokine expression due to SAP, may help to downregulate the response of the innate immune system.

However, CD206 (MR receptor) was moderately upregulated in the presence of SAP. *Candida* binding to MR (CD206) stimulates macrophages to secrete the proinflammatory cytokine IL-17 (47), a feature we observed. The amount of IL-17 secreted was not altered by incubating the macrophages with SAP, but it was the only proinflammatory cytokine observed to increase in the presence of SAP and yeasts. Thus, we find that for invasive candidiasis, the presence of SAP on macrophages or yeasts, shifted the macrophage response toward an anti-inflammatory outcome.

### PMNs and SAP.

We did not find SAP-dependent differences in neutrophil phagocytosis of C. albicans. SAP reduces neutrophil adhesion and spreading (23), and this may have led to the very small numbers of yeasts phagocytosed.

### Conclusion.

**FIG 6:**
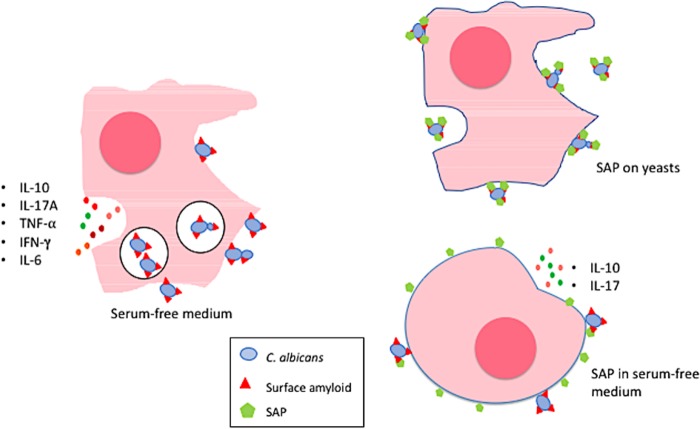
Figurative rendition of important findings of this investigation involving human macrophage-SAP-Candida albicans yeast interactions. (Left) The macrophage is a control macrophage (cultured in serum-free medium) and C. albicans yeasts added to it in serum-free medium. The cell secretes numerous cytokines and phagocytoses yeasts in great numbers. (Top right) The macrophage is cultured like the control macrophage, but the C. albicans yeasts are soaked in normal human serum or in purified SAP for an hour before addition to the macrophage, allowing SAP to bind to the fungal functional amyloid. Phagocytosis is reduced >80%. (Bottom right) The macrophage is cultured identically to the control macrophage except that it is incubated with SAP (30 µg/ml) for 1 h before C. albicans yeasts are added. Like the macrophage above it, there is a >80% reduction in phagocytosis of yeast cells, but only IL-10 and IL-17A are secreted in increased amounts. After SAP treatment, the macrophages were more rounded in their overall shape, a reflection of their quiescence.

SAP binds to C. albicans fungal cell surface functional amyloid, a structure recognized by macrophages. SAP bound to fungal surfaces is antiphagocytic and anti-inflammatory, reducing phagocytosis of fungi and increasing IL-10 secretion (Fig. 6). Although the question remains open whether SAP binding to fungi helps the host or the pathogen (5, 48), it appears from our work that SAP-coated yeasts are either masked from the innate immune system or, upon contact with host cells, dampen the immune response.

## MATERIALS AND METHODS

### Macrophages.

Human macrophages were isolated from whole blood using the SepMate-50 system (Stemcell Technologies, Seattle, WA) and following a University of Arizona IRB-approved protocol with written consent from the donors. After isolation, the cells were cryopreserved gradually with a controlled rate freezer. For experimental use, cryopreserved peripheral blood mononuclear cells (PBMCs) from normal (healthy) adult controls were placed in complete RPMI (RPMI, 10% fetal bovine serum, penicillin/streptomycin, and DNase [3 ng/ml]) and thawed to 37°C. Cells were collected by centrifugation at 500 × *g* for 6 min, resuspended in X-VIVO-15 (Lonza, Walkersville, MD), a serum-free medium, plated into 96-well plates (Fisher Scientific) (10^5^ cells in 100 µl medium/well), and placed in an incubator overnight at 37°C with 5% CO_2_. The following day PBMCs were washed with serum-free medium and resuspended in fresh medium, human MCSF (Sigma-Aldrich, St. Louis, MO) was added to each well (1 ng/ml), and cells were incubated for 5 days at 37°C with 5% CO_2_. Experiments were also carried out in four-well slide chambers (Bio Express, Visalia, CA) with the same medium and additives and 200 µl/well. Slide chambers allowed for better photomicroscopy of the macrophage-fungus interactions.

### Fungi.

Two Candida albicans strains were used in this study, one a clinical isolate (Banner University Medical Center, Tucson, AZ) and the other, a quality control strain from the Clinical and Laboratory Standards Institute (Wayne, PA). There was no difference in results with the two strains. The two strains were maintained on YPD agar (RPI, Mount Pleasant, IL). For experiments, a loopful of fungi was added to 5 ml YPD broth (Life Technologies Corp., Carlsbad, CA) and incubated overnight at 26°C with shaking. After 24 h, the yeasts were collected by centrifugation at 1,200 × *g* for 5 min, resuspended, and washed thoroughly in Tris-buffered saline with 2 mM Ca^2+^ (TBS-C). This was repeated three times, and cells were then resuspended to 10^9^ cells/ml in TBS-C and used as described below. Saccharomyces cerevisiae strains Als5p, V326N, and EV (background W303-1B) expressing variations of the C. albicans Als5p protein (19) were under a galactose promoter; therefore, the strains were grown in CSM-Trp galactose broth plus adenine (Thermo Fisher, Waltham, MA). Yeast strains were maintained on agar with the same medium. S. cerevisiae strains were cultured in CSM-Trp galactose broth plus adenine at 26°C for 48 h, and washed with TBS-C as described above.

### Macrophage-yeast interactions.

Wells containing 5-day-old macrophages in chamber slides or 96-well plates were washed with serum-free medium, and 100 µl of fresh serum-free medium and 1 µl containing 10^6^ previously washed yeasts suspended in TBS-C were added to each well. For other experiments, alongside the addition of C. albicans alone to wells or chambers, C. albicans was also incubated in 100% normal human male AB serum (Innovative Research, Novi, MI) or SAP (Millipore, Temecula, CA) at 30 µg/ml for 1 h at 4°C, washed with TBS-C, resuspended in serum-free medium, and added to wells or chambers. The cocultures were incubated at 37°C on a rocking shaker for 30 min. The yeast/macrophage ratio varied from 100 to 10:1. A 30-min incubation of yeasts with macrophages was employed to minimize the number of yeasts forming germ tubes. Following incubation of yeasts with macrophages, the wells were washed gently three times with TBS-C, and the wells or slides were stained with Wright-Giemsa. Stained wells and slides were observed by light microscopy, and a binding index (number of yeasts bound/50 macrophages/well or chamber) and a phagocytic index (number of yeasts phagocytosed/50 macrophages/well or chamber) was determined for each replicate in 96 wells or chamber slides (49). Each assay was repeated a minimum of three times. More than 30 different cell donors were used, and there was a remarkable consistency in the numbers of yeast cells phagocytosed per macrophage whether the cells were from male or female donors or from one week to the next with the same donor.

### SAP and other additives.

Human SAP or buffer-exchanged SAP (as described by Shao et al. [22]) in serum-free medium was added to wells to a final concentration of 30 µg/ml (the normal level in human serum). Other additives included CRP (Fitzgerald Industries, Acton, MA), D-mannose (Sigma-Aldrich), EDTA (Sigma-Aldrich), normal human male AB serum (Innovative Research), or bovine serum albumin (BSA) (Sigma-Aldrich) as described in the Results.

### Flow cytometry.

Fungi were cultured overnight (C. albicans) or for 48 h (S. cerevisiae) as described above, collected by centrifugation, and resuspended in TBS-C. Cells were incubated in undiluted normal human male AB serum or in TBS-C with 30 µg/ml SAP for 1 h at 4°C, washed three times by centrifugation, and resuspended in TBS-C. Cells were then incubated in TBS-C with 1:100 rabbit polyclonal anti-human SAP antibodies (PA5-24171; Invitrogen), or 1:100 anti-human CRP mouse monoclonal antibodies (MABF1070; clone 2A8.1, EMD-Millipore Corporation) for 30 min at 4°C. Yeasts were then washed three times and incubated with 1:200 fluorescein-labeled goat anti-rabbit or anti-mouse antibody (Invitrogen) for 30 min, washed three times, and resuspended in TBS. Fluorescence of cells was read on a BD LSR II flow cytometer (BD Biosciences, San Jose, CA) and then analyzed using Flowjo version 10.

### Determination of macrophage antigen expression by immunocytochemistry.

Macrophages were cultured in serum-free medium in 96-well plates as described above. After 5 days, the medium was replaced with the following: fresh serum-free medium for 30 or 60 min (control); serum-free medium plus C. albicans yeasts for 30 min; serum-free medium plus SAP (30 µg/ml) for 1 h; serum-free medium plus SAP (30 µg/ml) for 1 h followed by C. albicans yeasts for 30 min; or serum-free medium with C. albicans yeasts that had been soaked in normal human serum at 4°C for 30 min. For staining, macrophages were fixed with acetone for 15 min and air dried for 15 min, and nonspecific binding was blocked by incubation in PBS containing 4% BSA (PBS-BSA) for 60 min (22). Wells were then incubated with 5 µg/ml primary antibodies in PBS-BSA for 60 min as previously described (38). Isotype-matched irrelevant antibodies were used as controls. Antibodies from BioLegend (San Diego, CA) were anti-CD45, mouse IgG1, (catalog no. 304002), anti-CD54 (ICAM), mouse IgG1 (catalog no. 353102), and anti-CD206, mouse IgG1 (catalog no. 321102). Antibodies from Abcam (Cambridge, UK) were anti-CD209, mouse IgG1 (ab89186), anti-IRF5, rabbit monoclonal (EPR6094), and anti-IFR4, rabbit monoclonal (EP5699). Irrelevant mouse IgG1 (BioLegend) or rabbit polyclonal antibodies (R&D Systems, Minneapolis, MN) at 5 µg/ml in PBS-BSA were used as controls. Primary antibodies were detected with either biotinylated donkey F(ab′)_2_ anti-mouse IgG or biotinylated donkey F(ab′)_2_ anti-rabbit IgG (all cross-adsorbed against human Ig; Jackson ImmunoResearch, West Grove, PA). All secondary antibodies were used at 1 µg/ml in PBS-BSA for 30 min. Biotinylated antibodies were detected by a 1/500 dilution of ExtrAvidin alkaline phosphatase (Vector Laboratories, Burlingame, CA) in PBS-BSA. Staining was developed with the Vector Red Alkaline Phosphatase kit (Vector Laboratories) for 5 to 7 min and then counterstained with Gill’s hematoxylin number 3 (Sigma-Aldrich) following the manufacturer’s directions. Macrophages were identified as 15- to 40-µm-diameter cells with a large nucleus and pronounced cytoplasm. Cells were washed, dried, and later rehydrated with water, and cells were counted microscopically. There were six wells of macrophages for each antibody, and the experiment was repeated twice (100 macrophages were counted per well).

### Polymorphonuclear leukocyte phagocytosis of yeasts.

Blood samples from five healthy adult volunteers (three women and two men) were drawn into vacutainer tubes containing EDTA (Fisher Scientific, Waltham, MA) with written approval following a University of Arizona IRB-approved protocol. The blood was layered onto Polymorphoprep (Thermo Fisher) in a polypropylene tube and processed according to the manufacturer’s directions and centrifuged at 500 × *g* for 30 min. Isolated polymorphonuclear leukocytes (PMNs) were washed with TBS-C by centrifugation at 500 × *g* for 5 min twice and resuspended in TBS-C to 10^7^cells/ml. Then 100 µl was placed in each well of 96-well plates, incubated with 30 µg/ml SAP or without SAP for 1 h at 37°C, and washed with buffer. Yeasts were added at a ratio of 100 to 10 yeasts/PMN or yeasts incubated in serum for 1 h were added to PMNs and incubated for 30 min at 37°C with shaking. The cells were washed three times with TBS-C and stained with Wright-Giemsa. The number of yeasts phagocytosed per 50 PMNs was determined by microscopy.

### Cytokine measurements.

Macrophages were cultured in serum-free medium as described above in 96-well plates. After 5 days, the medium was replaced with the following: fresh serum-free medium; serum-free medium plus 30 µg/ml SAP (1 h); serum-free medium with C. albicans yeasts; or serum free-medium plus SAP, followed by addition of yeasts for 30 min. Media were aspirated from the wells at various time points and stored at −20^°^C. Thawed media were assayed for human IL-6, TNF-α, IFN-γ, IL-10, and IL-17A homodimers using Ready-SET-Go ELISA kits following the manufacturer’s protocols (Invitrogen).

### Statistics.

All population totals were graphed using median values and 95% confidence intervals or means with standard errors. An unpaired, nonparametric Mann-Whitney test or a basic unpaired *t* test was used to determine statistical significance between different populations and treatment conditions. Medians are shown in some graphs to show the central tendency. Software used was Prism version 7 (Graphpad, San Diego, CA).
