# Supplementary material for: Serum Amyloid P Component Binds Fungal Surface Amyloid and Decreases Human Macrophage Phagocytosis and Secretion of Inflammatory Cytokines
Source: mBio. 2019 Mar 12;10(2):e00218-19. doi: 10.1128/mBio.00218-19 (PMC6414697; doi:10.1128/mBio.00218-19)
Supplement: FIG S1 [file mBio.00218-19-sf001.docx]

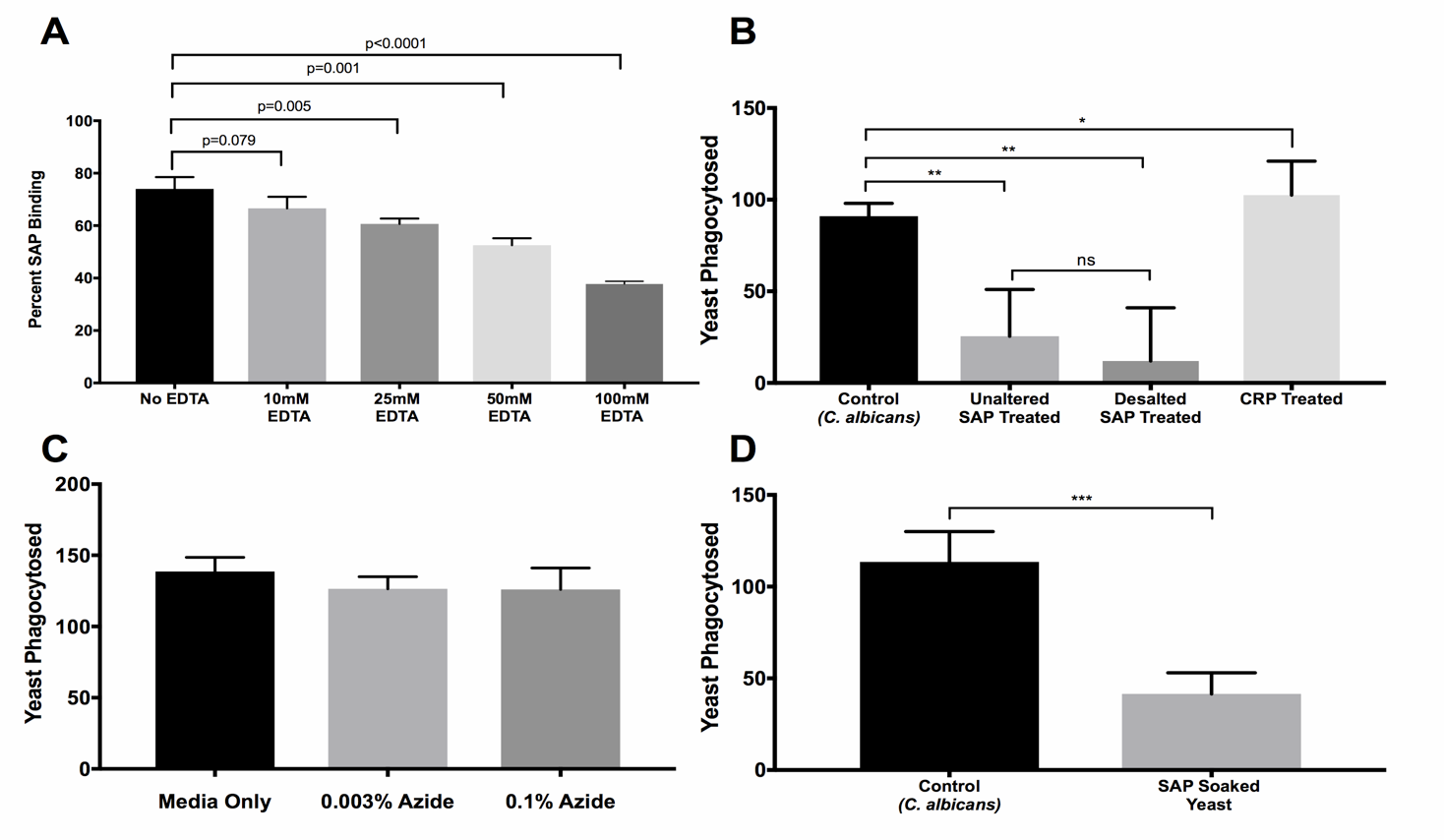


**Supplementary Figure 1:** Phagocytosis of *C. albicans* yeasts by human macrophages under different treatment conditions. **A.** Binding of SAP to C. *albicans* in the presence of the indicated concentrations of EDTA (flow cytometry). **B.** Phagocytosis of *C. albicans* by control macrophages; macrophages pre-treated with unaltered SAP (30 µg/mL); macrophages pre-treated with desalted SAP (SAP that had been purified by removal of trace azide, EDTA and NaCl); or macrophages pre-treated with 50 µg/mL C-reactive protein (CRP). **C.** Phagocytosis of *C. albicans* by macrophages in serum-free medium only, medium containing 0.003% sodium azide, or medium containing 0.1% sodium azide. **D**. Phagocytosis of untreated *C. albicans* by macrophages in serum-free media or phagocytosis of *C. albicans* pre-soaked in SAP (30 µg/mL, in TBS-C) for 1 hour, washed and added to macrophages in serum-free media.
